# The genetic Creutzfeldt-Jakob disease with E200K mutation: analysis of clinical, genetic and laboratory features of 30 Chinese patients

**DOI:** 10.1038/s41598-019-38520-y

**Published:** 2019-02-12

**Authors:** Li-Ping Gao, Qi Shi, Kang Xiao, Jing Wang, Wei Zhou, Cao Chen, Xiao-Ping Dong

**Affiliations:** 1 State Key Laboratory for Infectious Disease Prevention and Control, Collaborative Innovation Center for Diagnosis and Treatment of Infectious Diseases (Zhejiang University), National Institute for Viral Disease Control and Prevention, Chinese Center for Disease Control and Prevention, Chang-Bai Rd 155, Beijing, 102206 China; 2 Center of Global Public Health, Chinese Center for Disease Control and Prevention, Chang-Bai Rd 155, Beijing, 102206 China

## Abstract

Genetic Creutzfeldt-Jakob disease (gCJD) with E200K mutation is one of the common subtypes of human genetic prion diseases worldwide. In this study, we systematically analyzed 30 Chinese E200K gCJD cases for their epidemiological, clinical, laboratory and genetic features. The patients came from 12 different provinces, majority in northern part of China. The onset age varied from 42 to 71 year-old (y), with the median of was 57 y. The *CYP4X1* gene rs9793471 SNP was tested. Only one patient’s rs9793471 genotype was GA and the others’ were AA. The gender ratio (M: F) was 1:1.73 (11:19). The foremost symptoms and clinical progression of Chinese E200K gCJD patients were quite similar as sporadic CJD cases. Only a few cases (4/30) recalled clearly disease related family history. 74.1% (20/27), 86.7% (26/30) and 50.0% (13/26) of the cases were CSF 14-3-3 positive, sCJD associated abnormalities on MRI and special PSWC on EEG, respectively. The median clinical duration was 9 months (varying from 2 to 26 months). All 30 Chinese E200K gCJD patients were M129M and E219E homozygous. 21 members from 3 families conducted *PRNP* sequencing and 16 asymptomatic carriers of E200K mutation with M129M and E219E homozygous were identified. This is the largest study on E200K gCJD patients in China, which would benefit to the knowledge of E200K gCJD.

## Introduction

Human prion diseases are a group of rare, fatal and transmissible neurodegenerative diseases, including Kuru, Creutzfeldt-Jakob disease (CJD), Gerstmann-Sträussler-Scheinker syndrome (GSS) and fatal familial insomnia (FFI). The etiology of prion disease is known as a conformational change of the normal prion protein (PrP^C^) into a pathological conformation (PrP^Sc^) that aggregates in the central nervous system (CNS)^1^. CJD consists of three main catalogues: sporadic, familial, variant and iatrogenic forms. Familial/genetic CJD (fCJD/gCJD) accounts for about 10–15% of CJD cases worldwide^2,3^, which closely links to various pathogenic mutations in the prion protein-encoding gene, *PRNP*. Up to now, about 50 different pathogenic mutations in *PRNP* have been addressed, which result in dominant hereditary prion diseases^4,5^.

E200K gCJD, with the substation of Glutamate for Lysine at codon 200, is the most prevalent gCJD in many European countries^4,6^. According to the surveillance data from Chinese CJD Surveillance Center, E200K gCJD is the third most common genetic prion disease in Chinese, after D178N FFI and T188K gCJD^7–9^. In this prospective study, the epidemiological, clinical, genetic and laboratory characteristics of 30 Chinese E200K gCJD cases were comparatively investigated based on the onset age and gender. The main examinations and laboratory data were also proposed.

## Results

### General information

**Figure 1:**
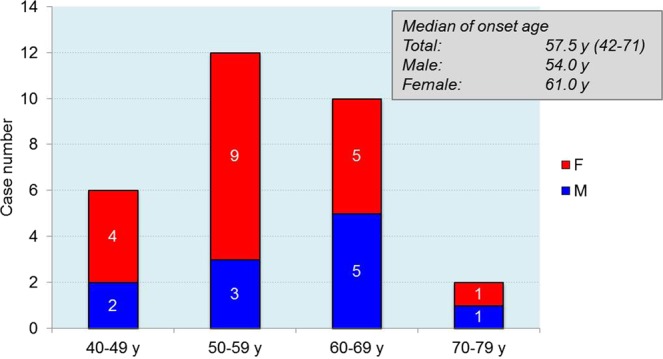
Onset-age distributions of 30 Chinese E200K gCJD patients. The medians of the onset age in total and in different genders are shown in the top right. F: female; M: male.

The first Chinese E200K gCJD case was described in 2008^10^. Up to April 2018, 30 E200K gCJD cases were identified and diagnosed by China CJD Surveillance Center, with 29 Han-Chinese and one Dong-Chinese. Analysis of the permanent addresses of the patients showed that they came from 12 provinces. 7 cases were from Tianjin (TJ), 5 from Hebei (HB), 4 from Henan (HN), 2 from Beijing (BJ), Zhejiang (ZJ), Neimenggu (NMG), Shandong (SD), and Jiangxi (JX), 1 from Guangdong (GD), Guizhou (GZ), Jilin (JL), and Heilongjiang (HLJ), respectively. The gender ratio (M:F) was 1:1.73 (11:19 cases). The onset ages varied from 42 to 71 year-old (y), with the median of 57.5 y. Most of the patients (12 cases) were in the group of 50–59 y, followed by the group of 60–69 y (10 cases). Female patients showed slightly younger onset age (median: 54 y) than male (median: 61 y), but without significant difference (Fig. 1).

### Clinical features

The interval times of those cases from onset to the reporting to CJD surveillance center varied from 2 to 24 months, with the median of 4.5 months. 12 out 30 cases were referred to the CJD surveillance center within 3 months after onset. The main clinical and laboratory features of those patients were summarized in Table 1. Majority of the patients displayed more than one initial symptom. Progressive dementia and recognition problems were mostly described, which appeared in 73.3% (22/30) cases. Various mental problems were recorded in 16 cases, followed by pyramidal and extrapyramidal symptoms in 9 cases, cerebellum symptoms in 6 cases, and sleep disorder in 3 cases. Along with the disease progression, more sporadic CJD (sCJD) associated signs appeared. In the context of whole clinical courses, dementia was noticed in 96.7% (29/30) patients, pyramidal or extrapyramidal dysfunction in 73.3% (22/30), myoclonus in 70.0% (21/30), visual or cerebellar disturbance in 83.3% (25/30) and akinetic mutism in 53.3% (16/30). The patients were further grouped based on gender and onset age (<60 y and ≥60 y). As shown in Table 1, female patients seemed to be less frequent having cerebellum disorders (P = 0.0156) as the foremost symptoms as male. The older patients appeared higher ratio having cerebellum disorders, although without statistical difference. For the major sCJD associated neurological signs during the clinical course, there was no statistical difference between the groups of gender and onset-age. The older patients seemed to display higher ratios having more symptoms than young patients, especially mutism at later stage of diseases. Myoclonus, pyramidal and extrapyramidal symptoms were slightly frequent in male patients.

**Table 1:** The main clinical and laboratory features of Chinese E200K gCJD patients.

Items	Total (n = 30)	Gender			Age of onset		
		M (n = 11)	F (n = 19)	P value	<60 y (n = 18)	≥60 y (n = 12)	P value
Age of onset (median)	57.5 y	61 y	54 y	—	—		
**Foremost symptoms**							
Progressive dementia	22 (73.3%)	8 (72.7%)	14 (73.7%)	1.0000	12 (66.7%)	10 (83.3%)	0.4192
Mental problems	16 (53.3%)	5 (45.5%)	11 (57.9%)	0.7065	10 (55.6%)	6 (50.0%)	1.0000
Cerebellum symptoms	9 (30.0%)	5 (45.5%)	1 (5.3%)	0.0156	2 (11.1%)	4 (33.3%)	0.1844
Pyramidal & extrapyramidal symptoms	6 (20.0%)	4 (36.4%)	5 (26.3%)	0.6871	4 (22.2%)	5 (41.7%)	0.4181
Sleeping disturbances	3 (10.0%)	1 (9.1%)	2 (10.5%)	1.0000	1 (5.6%)	2 (16.7%)	0.5478
**Major symptoms**							
Dementia	29 (96.7%)	10 (90.9%)	19 (100.0%)	0.3667	17 (97.4%)	12 (100.0%)	1.0000
Myoclonus	22 (73.3%)	9 (81.8%)	13 (68.4%)	0.6722	13 (72.2%)	9 (75.0%)	1.0000
Visual or cerebellar disturbance	21 (70.0%)	9 (81.8%)	12 (63.2%)	0.4189	12 (66.7%)	9 (75.0%)	0.7036
Pyramidal and extrapyramidal symptoms	25 (83.3%)	9 (81.8%)	16 (84.2%)	1.0000	13 (72.2%)	12 (100.0%)	0.0657
Mutism	16 (53.3%)	5 (45.5%)	11 (57.9%)	0.7065	8 (44.4%)	8 (66.7%)	0.2839
**Examinations**							
14-3-3^a^	20 (74.0%)	8 (80.0%)	12 (70.6%)	0.6784	12 (75.0%)	8 (72.7%)	1.0000
EEG^b^	13 (50.0%)^b^	4 (40.0%)	9 (56.3%)	0.6882	8 (50.0%)	5 (50.0%)	1.0000
MRI	26 (86.9%)	9 (81.8%)	17 (89.5%)	0.6111	15 (83.3%)	11 (91.7%)	1.0000

^a^Tested numbers for 14-3-3: 27.

^b^Examined numbers for EEG: 26.

### Clinical examination and laboratory tests

Lumber puncture was performed in 27 patients and the routine biochemistry items were all in the normal ranges. Western blot for CSF 14-3-3 was positive in 74.3% (20/27) cases. There was no significant difference in CSF 14-3-3 positive between genders or between young and older patients. Like the observation in sCJD patients, E200K gCJD cases with CSF 14-3-3 positive seemed to have more clinical manifestations. Half of the patients (10/20, 50.0%) of 14-3-3 positive displayed four and more than four sCJD-related symptoms, while 6 out of 7 patients who were 14-3-3 negative showed three and less three signs during their clinical courses.

EEG was recorded in the 26 cases, half (13/26) of which revealed sCJD associated periodic sharp wave complexes (PSWCs). Female patients appeared higher ratio of PSWC positive. All cases received MRI scanning, 26 showed abnormalities. Elder patients seemed to have higher rate of MRI typical abnormality, which was up to 100% in the group of 60–69 y. Additionally, 9 cases showed all positive on CSF-14–3–3, special abnormalities on MRI and PSWC on EEG. 95.0% (19/20) of the cases with CSF 14-3-3 positive displayed special abnormality on MRI. 84.6% (11/13) of the cases with PSWC on EEG showed abnormalities on MRI. Among the 11 cases with PSWC on EEG who performed CSF 14-3-3 Western blots, 9 cases (81.8%) showed CSF 14-3-3 positive. Only one case was negative in all three examinations.

### Survival time

**Figure 2:**
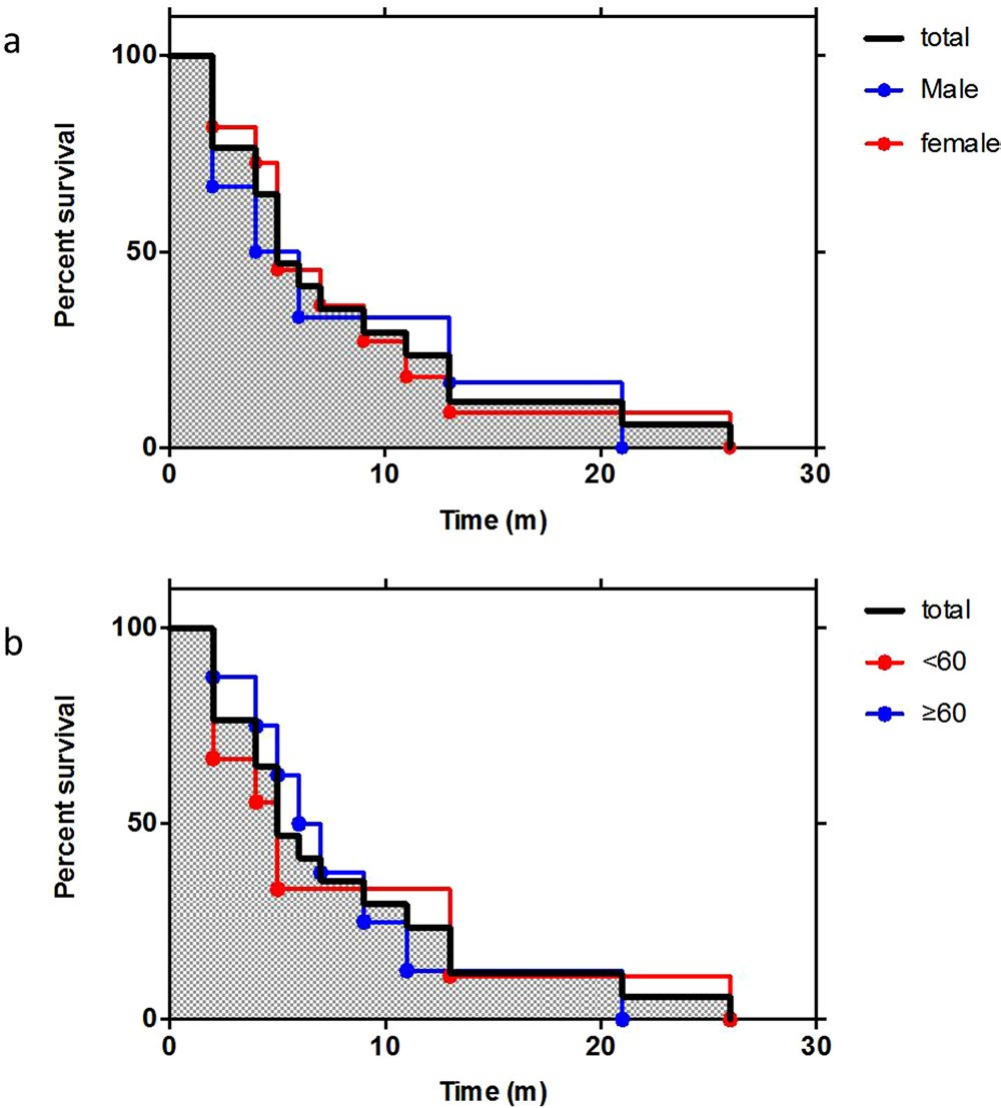
The curves of the survival times of 30 E200K gCJD patients based on different groups. (**a**) male and female patients; (**b**) <60 or ≥60 y at onset.

By the end of April 2018, 21 out of 30 E200K gCJD patients died, 6 lost contact and 3 are still alive. The clinical durations of the dead patients varied from 2 to 26 months, with the median of 9 months. 57.1% of the cases (12/21) died within one year after onset, and 47.6% (10/21) died within 6 months after onset. One patient lived longer than two years. The survivorship curve displayed as Fig. 2. Male patients (median: 8 m) seemed to have shorter duration than female (median: 9.7 m). There was no significant difference in the clinical duration between young and older patients, between the patients with more (≥four) and less (≤three) sCJD-related symptoms, with CSF 14-3-3 positive, PSWC in EEG and typical abnormalities in MRI.

### *PRNP, CYP4X1* rs9793471 sequencing and family history

**Figure 3:**
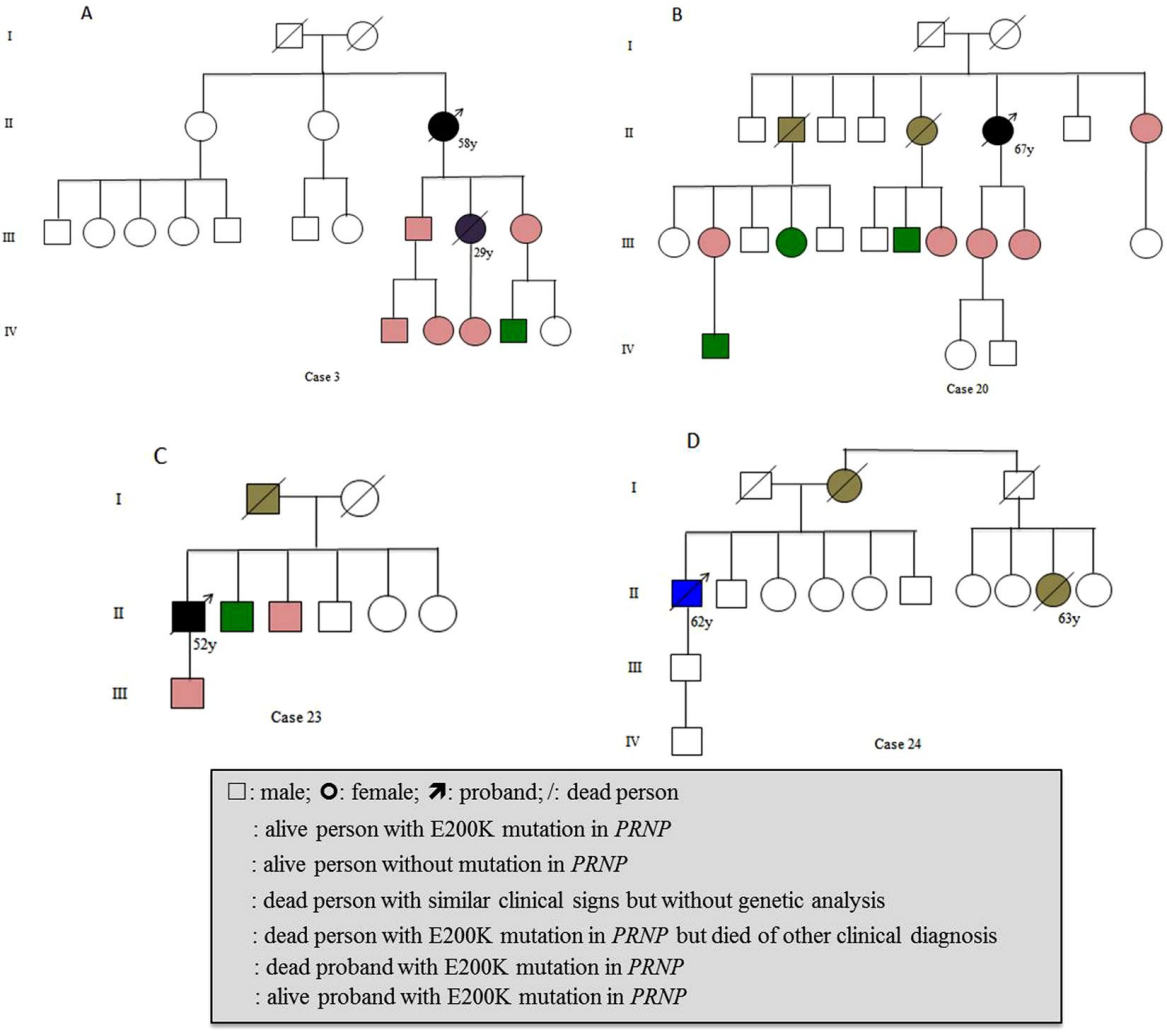
Disease related family histories and E200K mutations within *PRNP* in four families. (**A**) Case 3; (**B**) Case 20; (**C**). Case 23; (**D**) Case 24. The significances of various shapes, lines and colors are summarized in the panel below.

All 30 E200K gCJD patients were confirmed to have a mutation with the substation of Glutamate for Lysine at codon 200 in one of the *PRNP* allele. All cases were methionine homozygous genotype at codon 129 (M129M) and glutamic acid homozygous at codon 219 (E219E) of *PRNP*. Twenty nine cases were *CYP4X1* rs9793471 AA genotype, and one case was GA. The number was too small to analyze the correlation between rs9793471 genotype and onset age. Only four cases recorded possible disease-related family history. Case 3 was 58 year-old women, who showed progressive dementia, cerebellar disturbance and akinetic mutism at the end stage. She died in 5 months afterwards. Her son, elder daughter, young daughter and four grandkids donated their blood samples for *PRNP* sequencing. Six of them were E200K mutation carries. Her elder daughter died of sudden cerebral hemorrhage at the age of 29 year-old three years after *PRNP* sequencing (Fig. 3A). Case 20 appeared neurological symptoms at 67 year-old. Her elder sister was recalled to die with the similar clinical manifestation but without referable neurological diagnosis. One of her elder brothers died at 62 year-old without definite diagnosis. The family member recalled that he had visual impairment and died a couple of months after onset. 7 members of this family were tested with *PRNP* sequencing. Her two daughters, her young sister and one niece were E200K mutation carries. The other three tested members, a nephew, a niece and a grandnephew, were wild-type *PRNP* (Fig. 3B). Case 23 was male who displayed clinical symptoms at 52 year-old. His father was recalled to die with the similar symptoms. *PRNP* sequencing revealed his son and one of his brothers were E200K mutation carries (Fig. 3C). Case 24 appeared neurological symptoms at 62 year-old. His mother was dead with similar clinical manifestation (Fig. 3D). The family members also recalled that one of his younger female cousins was dead of neurological problems. None of his family member donated the blood for *PRNP* sequencing. He is still alive, with the symptoms of dementia, pyramidal and extrapyramidal dysfunctions, and is now bedridden. In addition, all asymptomatic E200K carriers in this study remained healthy by the end of April, 2018.

## Discussion

In this study, we systematically analyzed 30 Chinese E200K gCJD cases for their epidemiological, clinical, laboratory and genetic features. From our surveillance data of human prion diseases since 2006, about 150 cases of genetic prion diseases have been identified, which contain 15 different subtypes of *PRNP* mutations, which accounts about 10% of all referring human prion diseases^7^. E200K gCJD is the third most commonly observed genetic prion disease in China after D178N FFI and T188K gCJD^7,8,11^. E200K mutation is the largest cluster of gCJD existing in the North African Jewish community of Libyan and Tunisian ancestry^12^. In many European countries E200K is the most prevalent mutation in Caucasian^4,13–16^. Among the 30 E200K gCJD cases in this study, the number of female patients is slightly more than that of male ones. All E200K gCJD cases in this study appear sporadic onset, without any relationship of consanguinity. Although the majority of those E200K gCJD cases are come from northern provinces of China, the relatively small numbers of the cases seems not to be suitable for further analysis of geography association.

The onset and clinical characteristics of Chinese E200K gCJD are more like as sCJD, although the onset age of E200K gCJD is younger than that of sCJD^8,17^. *CYP4X1* SNP rs9793471 has an influence on age at onset of disease in patients with E200K genetic and sporadic CJD^18^. In this study, twenty nine cases were *CYP4X1* rs9793471 AA genotype, and only one case was GA. So the number was too small to analyze the correlation between rs9793471 genotype and onset age. Progressive dementia and mental symptoms are the most reported foremost ones. Along with the disease progression, dementia and other main sCJD-associated neurological signs are frequently observable. Like the other studies^19–21^, three patients in this study complain sleeping problems as the foremost symptoms. The first Chinese E200K patient who was reported in 2010 also had sleeping disturbance as the initial symptom^10^. The positive rates of CSF 14-3-3, PSWC in EEG and special abnormalities in MRI of those E200K gCJD cases are similar as those of Chinese sCJD^8^. Therefore, without *PRNP* sequencing, it is almost impossible to distinguish E200K gCJD and sCJD clinically. The median survival time of E200K gCJD cases is longer than that of 257 Chinese sCJD patients (4.5 moths)^8^. However, similar as the data of sCJD, the exact clinical duration each case varies largely, while no special associations of the survival time with gender, onset age and other clinical features are addressed.

Clinically, the disease associated family history among 30 Chinese E200K gCJD patients is less frequent, that only four cases are recalled to have disease-associated family history during our follow-up survey. Several E200K mutation carries are identified in the three families having performed *PRNP* sequencing. It is reported that carriers of the E200K mutation are born with this dominant mutation in the *PRNP* gene, but most remain asymptomatic until middle age and some only develop the disease after the age of 80 years^22,23^. Although the clinical durations of three dead patients with positive family history seem to be slightly shorter, however, without showing significance by statistical analysis. Additionally, all 30 Chinese E200K gCJD patients are M129M and E219E homozygous, which reflects a typical profile of *PRNP* genotype of Northeastern Asian. Actually, all Chinese patients with various genetic prion diseases are M129M so far, whose rate is obviously higher than that of normal Chinese population (about 93%) and sCJD patients (98.5%)^8^.

The features of neuropathology and PrP^Sc^ deposit of Chinese E200K gCJD patients remain unknown, as none of those patients undergoes postmortem or biopsy assay. Typical spongiform changes and synaptic-type PrP^Sc^ deposition have been described in the brains of E200K gCJD patients in Japan, which resembles MM1-type sCJD^24^. A more detailed neuropathological study has proposed that the immunoreactivity of PrP of E200K gCJD cases, defined as multiple globular structures, is distinct from sCJD with intraneuronal small dot like profile^25^. On the other hand, higher positive rate of E200K gCJD patients in CSF RT-QuIC has been reported repeatedly^26–28^. Moreover, the CSF samples of E200K gCJD cases show higher maximal intensity of ThT fluorescent signal and shorter lag phase of positive conversion than those of sCJD ones. It possibly reflects more prion agent releasing from brain tissues to CSF. In line with those observations, higher ThT values and shorter lag phases in CSF RT-QuIC assays are also observed in Chinese E200K gCJD cases compared with the data of sCJD, as well as T188K gCJD and D178N FFI (unpublished data).

Nowadays, therapeutic intervention in human prion diseases is still unsuccessful. Some protocols have showed the abilities to reduce the speed of deterioration of prion disease in short periods of time, but none shows the effect to reverse the severe neurological deficits apparent already at diagnosis^29–31^. Several mouse models for human gCJD have been constructed, such as mouse models for E200K gCJD^32^, P102L GSS^33^, A117V GSS^34^ and D178N FFI^35^. Recently we have constructed a transgenic mice expressing human *PRNP* with T188K mutation for T188K gCJD (unpublished data). Using those mouse models, the pathogenesis of human genetic prion diseases and potential therapeutic and prophylactic tools will be further exploded.

## Materials and Methods

### Ethics statement

This study was approved by the Ethical Committee of the National Institute for Viral Disease Control and Prevention (Beijing, China) under the protocol 2011ZX10004-101. The written informed content of each patient was got either by a family member or a relative of the patient prior to inclusion in the study, according to the requirement of CJD surveillance. And all methods were performed in accordance with the relevant guidelines and regulations.

### Study population and data collection

30 genetic-confirmed E200K gCJD cases from Chinese CJD Surveillance Center from 2011 to 2018 were enrolled in this study. The diagnosis of CJD was based on the diagnostic criteria issued by China National Health Commission, requiring a progressive dementia with at least two out of four following manifestations clinically: myoclonus, visual or cerebellar symptoms, pyramidal or extrapyramidal dysfunction or akinetic mutism. Diagnosis of probable CJD was supported by typical changes in magnetic resonance imaging (MRI) with the presences of high signal in caudate/putamen and/or symmetrical or dissymmetrical cortical ribbon syndrome on diffusion-weighted imaging (DWI), or electroencephalography (EEG) with the presences of periodic sharp wave complexes (PSWC), or CSF 14-3-3 positive. Diagnosis of E200K gCJD needs the sequencing of *PRNP*. All patients were followed up till death by the specialist in Chinese CJD Surveillance Center. The survival time was evaluated from disease onset to death.

### Laboratory tests

The whole blood and CSF samples of the referred patients were collected by the clinicians from local hospitals according to the CJD surveillance protocol^7,17,36^. The whole DNAs of the blood samples were extracted by a commercial kit (Qiagen 51104). The *PRNP* gene was amplified by a PCR protocol with the specific primers (forward: 5-GGCAAACCTTGGATGCTGG-3, reverse: 5-CCCACTATCAGGAAGATGAGG-3) and subjected into sequencing analysis of *PRNP* and polymorphisms of codon 129 and 219^17,36^. The *CYP4X1* rs9793471 SNP was amplified and sequenced with specifically designed primers(forward: TGCGTTAACTTCACAAGGTGACA, reverse: GGTCAGGGCCTAATGATCCTCC) to test rs9793471 genotype. Then analyze the relation between *CYP4X1* and the age at onset. The CSF samples were employed into Western blot for 14-3-3 protein based the protocol described previously^8,17,36^.

### Statistical analysis

The statistical analyses were performed using the SAS 9.4 statistical software program. Significance was determined using fisher’s precise test. A difference was considered statistically significant if the *p* value was <0.05.
